# GCARDTI: Drug–target interaction prediction based on a hybrid mechanism in drug SELFIES

**DOI:** 10.1002/qub2.39

**Published:** 2024-04-01

**Authors:** Yinfei Feng, Yuanyuan Zhang, Zengqian Deng, Mimi Xiong

**Affiliations:** ^1^ School of Information and Control Engineering Qingdao University of Technology Qingdao China; ^2^ Qingdao Municipal Hospital Qingdao China

**Keywords:** drug–target interaction, drug SELFIES, hybrid mechanism, random forest

## Abstract

The prediction of the interaction between a drug and a target is the most critical issue in the fields of drug development and repurposing. However, there are still two challenges in current deep learning research: (i) the structural information of drug molecules is not fully explored in most drug target studies, and the previous drug SMILES does not correspond well to effective drug molecules and (ii) exploration of the potential relationship between drugs and targets is in need of improvement. In this work, we use a new and better representation of the effective molecular graph structure, SELFIES. We propose a hybrid mechanism framework based on convolutional neural network and graph attention network to capture multi‐view feature information of drug and target molecular structures, and we aim to enhance the ability to capture interaction sites between a drug and a target. In this study, our experiments using two different datasets show that the GCARDTI model outperforms a variety of different model algorithms on different metrics. We also demonstrate the accuracy of our model through two case studies.

## 1 INTRODUCTION

Drug–target interactions (DTIs) are a key issue in the development of new drugs and in drug repurposing [1]. The biological effect of a drug is the result of the reaction between small drug molecules and target macromolecules [2]. Although traditional biological experiments are reliable, it is very expensive and time‐consuming to test each drug target pair. According to statistics, it takes more than 10 years to develop a new drug from the development stage to the clinical use stage. Drug development is costly and time‐consuming, and drugs bring some toxic side effects to the human body. Fewer than 10% of new drugs are approved for clinical use. Therefore, researchers increasingly use computer methods to explore prediction of DTIs. As computer methods, DTIs’ prediction methods can be summarized into three categories: structure‐based methods [3, 4], ligand‐based methods [5] and machine learning‐based methods [6]. The structure‐based and ligand‐based methods have been widely used by researchers due to their good performance. However, structure‐based approaches require molecular docking simulations, which cannot be achieved without knowledge of the 3D structure of the target protein. The ligand‐based method requires new active molecule prediction for the same target protein based on the known bioactive molecules. If the number of known bioactive molecules is not enough, the prediction is poor.

In recent years, due to the great improvement of computing power and breakthroughs in machine learning algorithms [7], models based on machine learning have been welcomed by researchers. Compared with traditional molecular docking technology, machine learning can test a large amount of drug and target data in a relatively short time [8]. Machine learning can be roughly divided into three types: (1) feature vector‐based methods [9], which uses some machine learning methods such as support vector machine [10] and random forest [11] for prediction and classification by input sequence of the medicinal chemical structure descriptor and the target and (2) similarity‐based methods, which generate a similarity matrix for the drug and the target by calculating the chemical structure or properties of the drug and the target, respectively; these two similarity matrices have been used in many methods, including Laplacian regularized least squares (LapRLS) [12], bipartite local method [9], and Gaussian interaction spectrum [13], to explain the relationship between a drug and a target from chemical space and target space, respectively. (3) Other methods, for example, the use of biomedical literature [14] and pharmacological information of drugs [15], through text mining technology [16], to extract the implied relationship between drugs and targets. But a major drawback of textual relationships is that they may not represent true DTIs. These DTI prediction methods involve shallow learning methods that cannot fully extract the deep and complex associations between drugs and targets.

Deep learning algorithms are now also widely used in feature mapping [17], classification tasks [18] and disease prediction [19], so they promote the development of DTI predictions. Graph representation learning [20], as a form of deep learning, is also widely used in DTI prediction learning and can effectively combine the characteristic information and structural information of drugs and targets. The main and typical methods of representation learning based on graph structure include deep walk [21] and Node2vec [22]. These typical methods of representation learning based on graph features include the graph convolutional neural network (GCN) [23], GraphSAGE [24], and the graph attention network (GAT) [25]. The drug–disease association prediction based on the layer attention graph convolutional network proposed by Yu et al. [26] captures feature information from drug structure space and disease space through a graph GCN. The DTI predictions of the multi‐layer attention mechanism proposed by Zheng et al. achieve the fusion of different levels of attention features [27]. A heterogeneous network can be constructed to capture special associations between a drug and a target, and An et al. [28] have used a heterogeneous network embedding a framework for predicting similarity‐based DTIs. Some studies have used these deep learning methods to predict DTIs, almost all of which involve drug coding by using drug SMILES (Simplified Molecular Input Line Entry System). However, these simple methods have some shortcomings in graph information aggregation and have limitations in the capture of feature information of drug and target space. A single feature extractor cannot extract more complete and effective information from the structure of drugs and targets. SMILES, which has become a standard tool in computational chemistry, is still string‐based representation of molecular information, and a large fraction of the resulting SMILES strings do not correspond to valid molecules. They are either grammatically invalid and do not even fit the molecular graph, or they violate basic chemical rules, such as the maximum number of valence bonds between atoms. Krenn et al. have proposed Self‐referencing Embedded Strings (SELFIES), a 100% robust molecular string representation [29]. The proposed SELFIES provides researchers with new options for drug molecular data information.

**FIGURE 1:**
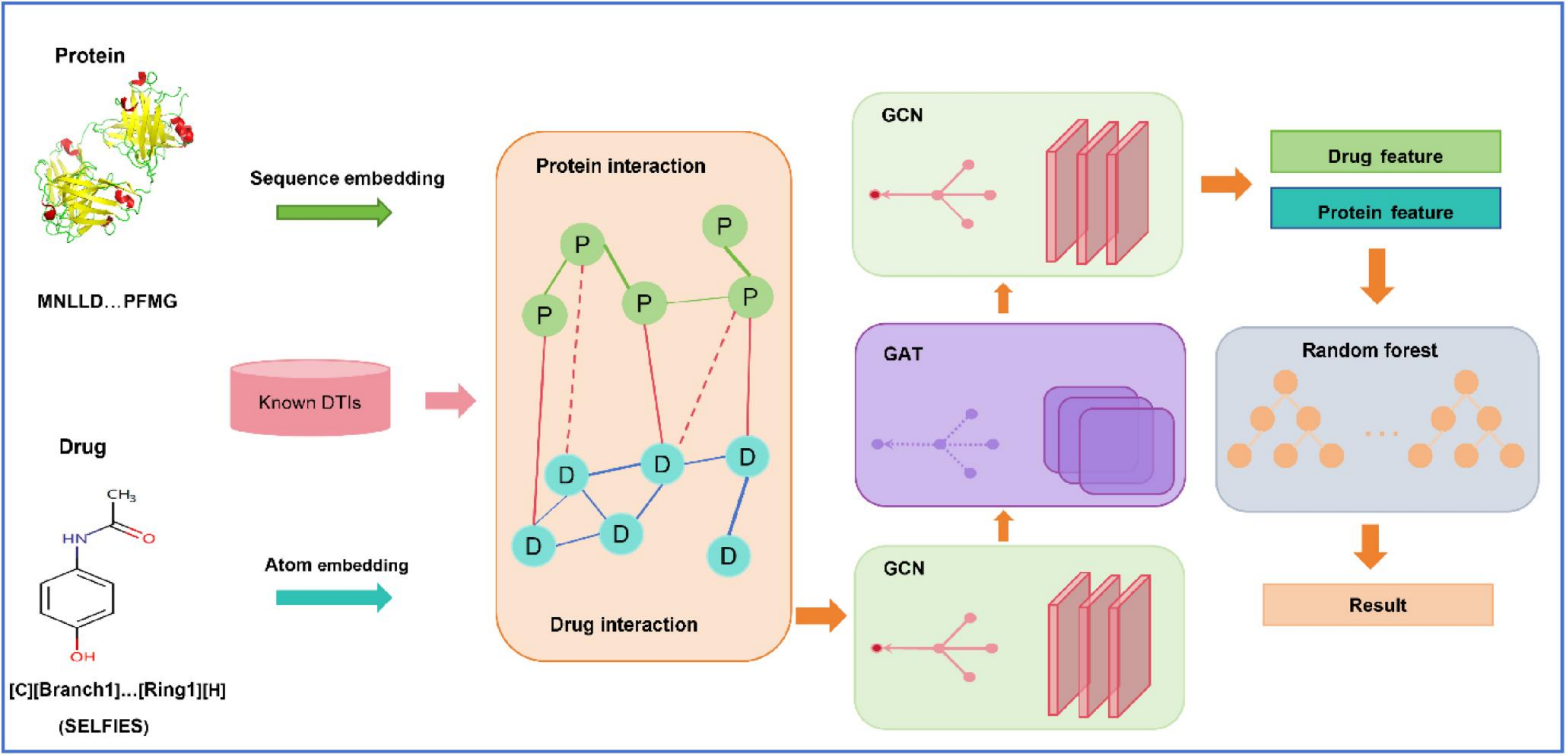
GCARDTI framework diagram: (1) Drug and target protein structures act as input, obtaining protein sequence and drug SELFIES representation, respectively, which are then vector embedded, and their heterogeneous network based on similarity and known association is constructed. (2) The network is input into a multi‐view feature extractor containing a hybrid mechanism of the GCN and the GAT to obtain low‐dimensional features. (3) Then, the network is put into the random forest for score prediction.

To address the shortcomings of the methods outlined above, we propose the GCARDTI model, as shown in Figure 1. SELFIES is a self‐referent embedded string that is 100% robust and uses drug molecular data that is different from previous ones. Each self‐embedded string corresponds to a valid molecule. SELFIES can be directly applied to any machine learning model without the need for model adaptation, and each generated candidate molecule is valid and corresponds well to each drug molecule structure. SELFIES combines the constructed heterogeneous network common to target sequences and feeds it into the hybrid mechanism of the GCN and the GAT to capture high‐dimensional features from both the pharmacochemical space and the target space. The GCN extends the convolution operator to irregular regions, specifically to update the atomic feature vectors according to their corresponding neighboring atoms connected by chemical bonds. This propagation mechanism captures the structural information of molecules. The GCARDTI model uses the GAT attention module to capture the contribution of substructures to each drug and target. Finally, a layer of GCN is used to aggregate the substructure information of each drug and target, to update the feature vectors of each drug and target, and finally obtain low‐dimensional and effective drug target feature information. The low‐dimensional feature representation is used as input to obtain better prediction and avoid the negative effects of feature dimension and feature information importance on predicted drugs and targets. Then, the random forest module is used to predict the score of feature representation, and the importance of feature and the interaction between different features can be judged. For imbalanced datasets, random forest can balance the error. If most features are lost, the accuracy can still be maintained, and the model has strong robustness and generalization ability. Using two datasets from different sources, we show that some metrics such as AUPR and AUROC of the GCARDTI model have some advantages over those of other methods.

## 2 RESULTS

In our experiments, we contrasted different types of model approaches using two different datasets, each processed consistently. The known DTIs were used as positive samples, and the remaining unknown associations were used as negative samples. Twenty percent of the positive and negative samples were randomly selected as the independent test set, and the remaining dataset samples were used as the cross‐validation set. The cross‐validation set was applied to 5‐fold cross‐validation, in which the positive samples were divided into five parts. In each part, a portion of the positive samples served as the validation set along with an equal number of negative samples randomly drawn from the cross‐validation set, and the remainder of the cross‐validation set served as the training set. To evaluate the accuracy of GCARDTI, the receiver operating characteristic (ROC) curve was used. It is plotted by two variables, the false positive rate and the true positive rate. Considering the bias performance of the area under the curve (AUC) of the imbalanced dataset, we also used the precision‐recall (PR) curve to accurately reflect the actual performance of the prediction model. ROC‐AUC and PR‐AUC, the areas under the ROC curve and under the PR curve, respectively, were used to quantitatively represent the performance of ROC‐AUC and PR‐AUC, respectively. In addition, recall, F1‐score, and accuracy were used to evaluate the overall performance of the prediction model from different perspectives.

One of the datasets was taken from HampDTI [30]. We compared the performance of GCARDTI with DTI prediction methods based on heterogeneous networks, including three classical shallow models: NetLapRLS [31], CMF [32] and DTInet [33]. These are two models with better performance at present, NeoDTI [34] and HampDTI.

The comparison of the results from ROC‐AUC and PR‐AUC curves of our GCARDTI model with those of the other five methods are presented in Table 1. The bold font in the table indicates the highest score in this indicator. In the crossover experiment, we selected the best score. It can be intuitively seen that our model had superior performance than other methods. On this cross‐validation dataset, the GCARDTI model performed best (ROC‐AUC = 0.9405 and PR‐AUC = 0.9532), better than HampDTI (ROC‐AUC = 0.9273 and PR‐AUC = 0.9263) for both ROC‐AUC and PR‐AUC, but lower in F1‐score. The accuracy and recall of our model were also higher than those of other methods. In general, our model was the best in terms of almost all indicators, performing well, allowing extraction of the feature attributes in the network structure, and using the hybrid mechanism to better extract the feature attributes from the drug and target protein space.

**TABLE 1:** The performance was evaluated on the cross‐validation set of dataset 1.

Method	ROC‐AUC	PR‐AUC	F1‐score	Accuracy	Recall
NetLapRLS	0.9074	0.9180	0.8388	0.8321	0.8730
CMF	0.9010	0.9247	0.8468	0.8487	0.8367
DTInet	0.8612	0.8861	0.7996	0.8029	0.7853
NeoDTI	0.9150	0.9173	0.8562	0.8550	0.8628
HampDTI	0.9273	0.9263	**0.8689**	0.8648	0.8986
GCARDTI	**0.9405**	**0.9532**	0.8613	**0.8818**	**0.9060**

*Note*: The black font is the highest value under several methods.

To further investigate the generalization performance of the prediction model, we performed an independent test set that evaluated the prediction power not used for model training and hyperparameter determination. For independent tests, the highest 10 values were obtained for each test index, and the average values of each set of 10 were then used as the results of independent tests. As shown in Table 2, GCARDTI had the best performance regarding most indicators, especially in the case of PR‐AUC, which was 3.4% higher than other benchmark methods on average. In F1‐score and recall, GCARDTI results were lower than those of HampDTI, but other scoring indicators were higher than in the case of all methods, thanks to the strong generalization ability of our model. These scores reflect the superiority of the hybrid mechanism of our model, which can better capture feature information from the drug and target space.

**TABLE 2:** The performance was evaluated on an independent test set of dataset 1.

Method	ROC‐AUC	PR‐AUC	F1‐score	Accuracy	Recall
NetLapRLS	0.9003	0.8993	0.8506	0.8515	0.8469
CMF	0.8970	0.9210	0.8461	0.8487	0.8313
DTInet	0.8505	0.8567	0.7928	0.7823	0.8333
NeoDTI	0.9057	0.9002	0.8518	0.8458	0.8839
HampDTI	0.9209	0.8972	**0.8824**	0.8776	**0.9193**
GCARDTI	**0.9342**	**0.9311**	0.8813	**0.8990**	0.9079

*Note*: The black font is the highest value under several methods.

We compared the datasets used by the model DTI‐HETA [35]. Our model, GCARDTI, compares the following different models, including HIN2VEC [36], EVENT2VEC [37], and HEER [38], which deals with node embedding and relationship prediction of heterogeneous graphs, and GATNE [39] and PGCN [40], which are models based on end‐to‐end training and prediction.

**FIGURE 2:**
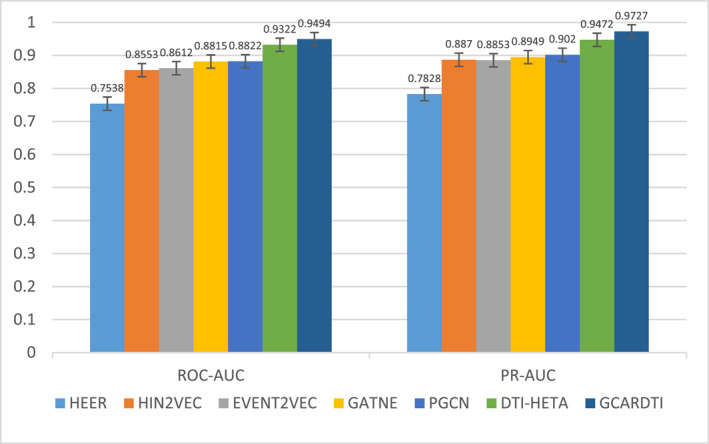
Comparison of predicted values of ROC‐AUC and PR‐AUC between the GCARDTI method and other six methods.

The comparison of predictions of ROC‐AUC and PR‐AUC between our model and the other six models in the second dataset is presented in Figure 2. It can be seen that our model had the highest scores (ROC‐AUC = 0.94 and PR‐AUC = 0.97). Compared with the second scoring DTI‐HETA model, the ROC‐AUC = 0.93 value was 1% higher, and the PR‐AUC = 0.94 value was 3% higher, confirming the good performance and reflecting the generalization of our model. The hybrid mechanism of our model can better capture feature information from the drug and target space.

### 2.1 Ablation experiments

**FIGURE 3:**
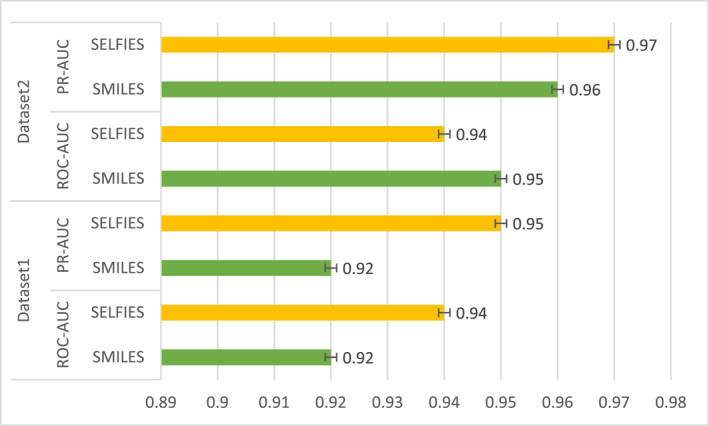
Evaluation plot of ROC‐AUC and PR‐AUC values of the GCARDTI method using drug SMILES and drug SELFIES.

In order to further confirm that the effect of the drug data SELFIES used in this paper is better than that of the conventional drug SMILES, we performed ablation experiments, under the same circumstances under other settings, on two different datasets. We, thus, compared the molecular string representation of the model GCARDTI using different datasets (Figure 3).

The ROC‐AUC and PR‐AUC values obtained from using drug SELFIES in dataset 1 were better than those from using drug SMILES in Figure 3. In the second dataset, the ROC‐AUC value of using SELFIES (ROC‐AUC = 0.94) was lower than that of using SMILES (SMILES = 0.95). The use of drug SELFIES gave results that were 1% higher than in the case of the use of the drug SMILES indicator, showing that the use of drug SELFIES can indeed improve the performance of the model and indicating that the use of drug SELFIES can better reflect the characteristics of the drug.

Two datasets were used to perform ablation experiments on the model to explore the influence of using different drug string codes and different GCN and GAT layers. As can be seen from Table 3, the performance of the model using drug SELFIES was better than that using SMILES. Although in dataset 2, the ROC‐AUC performance using SMILES was 1% higher than that using SELFIES, the other SELFIES indicators were superior to those of SMILES. We also did experiments using multi‐layer hybrid GCN. It can be clearly seen in the table that the model effect of multi‐layer hybrid mechanism was higher than that of single‐layer, indicating that the hybrid mechanism is a powerful means to capture the spatial characteristics of drugs and target proteins, and supporting the effectiveness and superiority of the structure of our model.

**TABLE 3:** Drug SELFIES or SMILES used on both datasets, *GAT* and *GCN* mixed.

Ablation tests	Dataset 1		Dataset 2	
	ROC‐AUC	PR‐AUC	ROC‐AUC	PR‐AUC
*GCN* + *GAT*_SMILES_	0.8421	0.8937	0.9125	0.9272
*GCN* + *GAT* + *GCN*_SMILES_	0.9236	0.9289	0.9566	0.9661
*GCN* + *GAT*_SELFIES_	0.9277	0.9301	0.9033	0.9365
*GCN* + *GAT* + *GCN*_SELFIES_	0.9405	0.9532	0.9494	0.9727

### 2.2 Case studies

**FIGURE 4:**
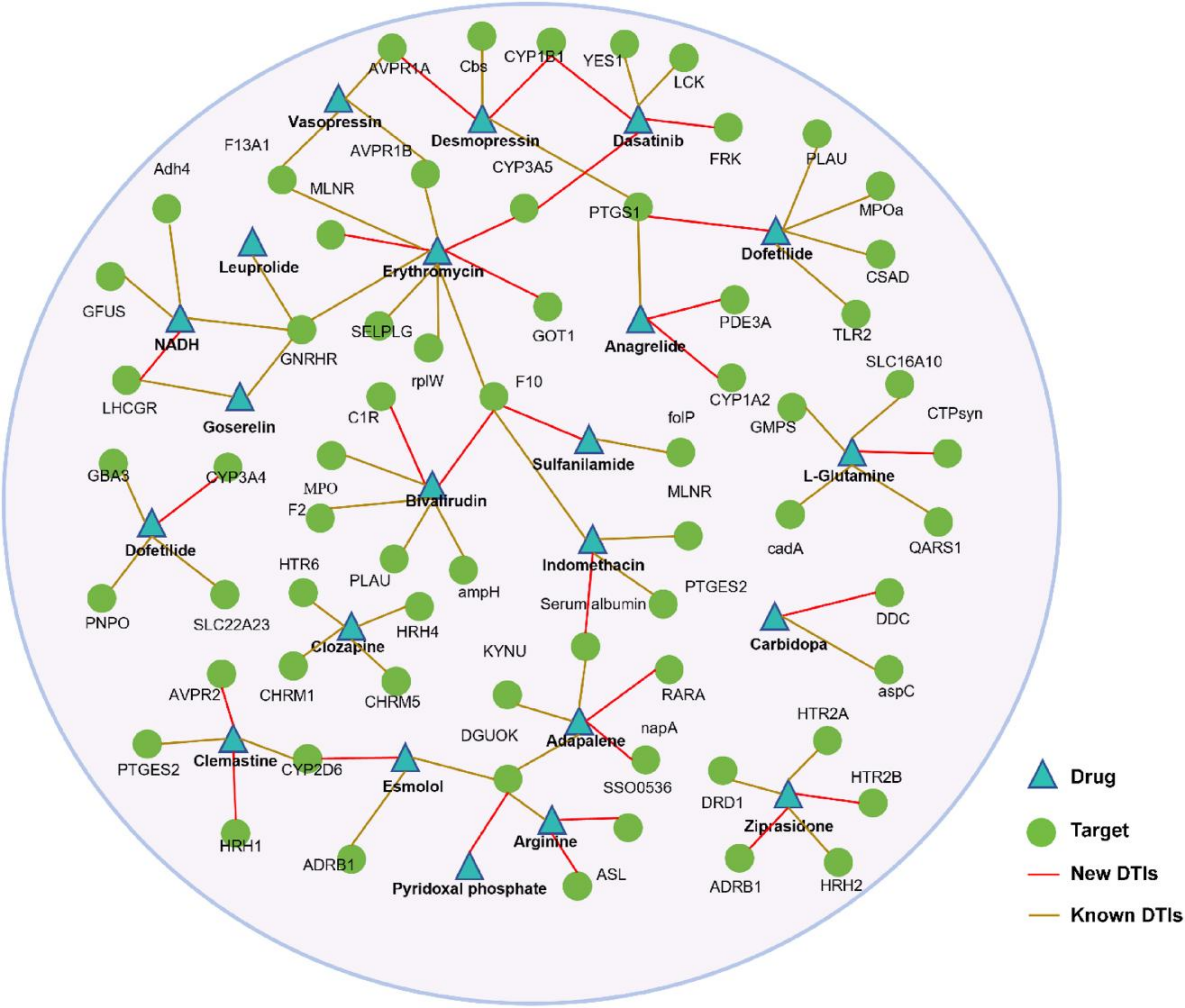
Network visualization of DTI of top 22 drugs predicted by the GCARDTI method. Target proteins are represented by green circles and drugs by blue triangles. The known DTIs are represented by orange lines, and the new predicted DTIs are represented by red lines.

In order to verify the predictive performance of the model, we studied the situation of 22 drugs in the top 50 scores predicted by the GCARDTI model, visualized as a network diagram (Figure 4) and selected several for case analysis.

Indomethacin, a derivative of indoleacetic acid, is a nonsteroidal anti‐inflammatory drug, which belongs to the antipyretic and analgesic drug category, and is suitable for antipyretic and inflammatory pain relief. Indomethacin was predicted to be associated with MLNR, PTGES2, and F10, and it was found to be associated with serum albumin through model prediction. The target was associated with adapalene. Cyclooxygenases can interact with its target receptors, serum albumin and the bile salt export pump. In our novel prediction, human cyclooxygenases were also found to be associated with these, and the accuracy of the prediction was verified by the DrugBank database. Dasatinib is a protein kinase inhibitor that inhibits BCR‐ABL kinase and SRC family kinases, as well as a number of other selective oncogenic kinases, including c‐KIT, ephrin (EPH) receptor kinase, and PDGFB receptor. Clinically, it mainly treats adult patients with Philadelphia chromosome positive (Pht) chronic myeloid leukemia in the chronic phase, the accelerated phase, and blast crisis (acute myeloid leukemia and ALL) who are resistant to or intolerant to imatinib mesylate. The predicted target tyrosine‐protein kinase FRK was associated with the drug, and this was verified from the DrugBank database. Through the above two cases, it can be found that our model GCARDTI predicts the accuracy of drug target proteins, which has important significance for the actual prediction of DTIs.

To further investigate the predictive power of the model GCARDTI in terms of DTIs, we conducted case studies of four drug candidates (ziprasidone, asenapine, quetiapine, and clozapine) in which we ranked the interaction scores of each drug candidate protein in descending order. The top six candidate proteins were analyzed as shown in Table 4. Among these 24 candidate proteins, they were included in DrugBank and DrugCentral, demonstrating the ability of GCARDTI to discover potential DTIs.

**TABLE 4:** The top 6 candidate proteins of four drugs.

Drug name	Rank	Target	Evidence
Ziprasidone	1	HTR2A	DrugCentral
	2	HTR2B	DrugCentral
	3	HRH2	DrugCentral
	4	ADRB1	DrugCentral
	5	ADRA2A	DrugBank
	6	DRD1	DrugCentral
Asenapine	1	CHRM4	DrugCentral
	2	CHARM1	DrugCentral
	3	PPARG	DrugCentral
	4	HTR2C	DrugBank
	5	HTR1A	DrugCentral
	6	ESR1	DrugCentral
Quetiapine	1	CHRM5	DrugBank
	2	CHRM4	DrugBank
	3	DRD5	DrugBank
	4	DRD2	DrugBank
	5	HTR2C	DrugBank
	6	HTR6	DrugBank
Clozapine	1	CHRM1	DrugBank
	2	HRH4	DrugBank
	3	HTR6	DrugBank
	4	CHRM5	DrugCentral
	5	HTR2C	DrugCentral
	6	ADRB1	DrugBank

**FIGURE 5:**
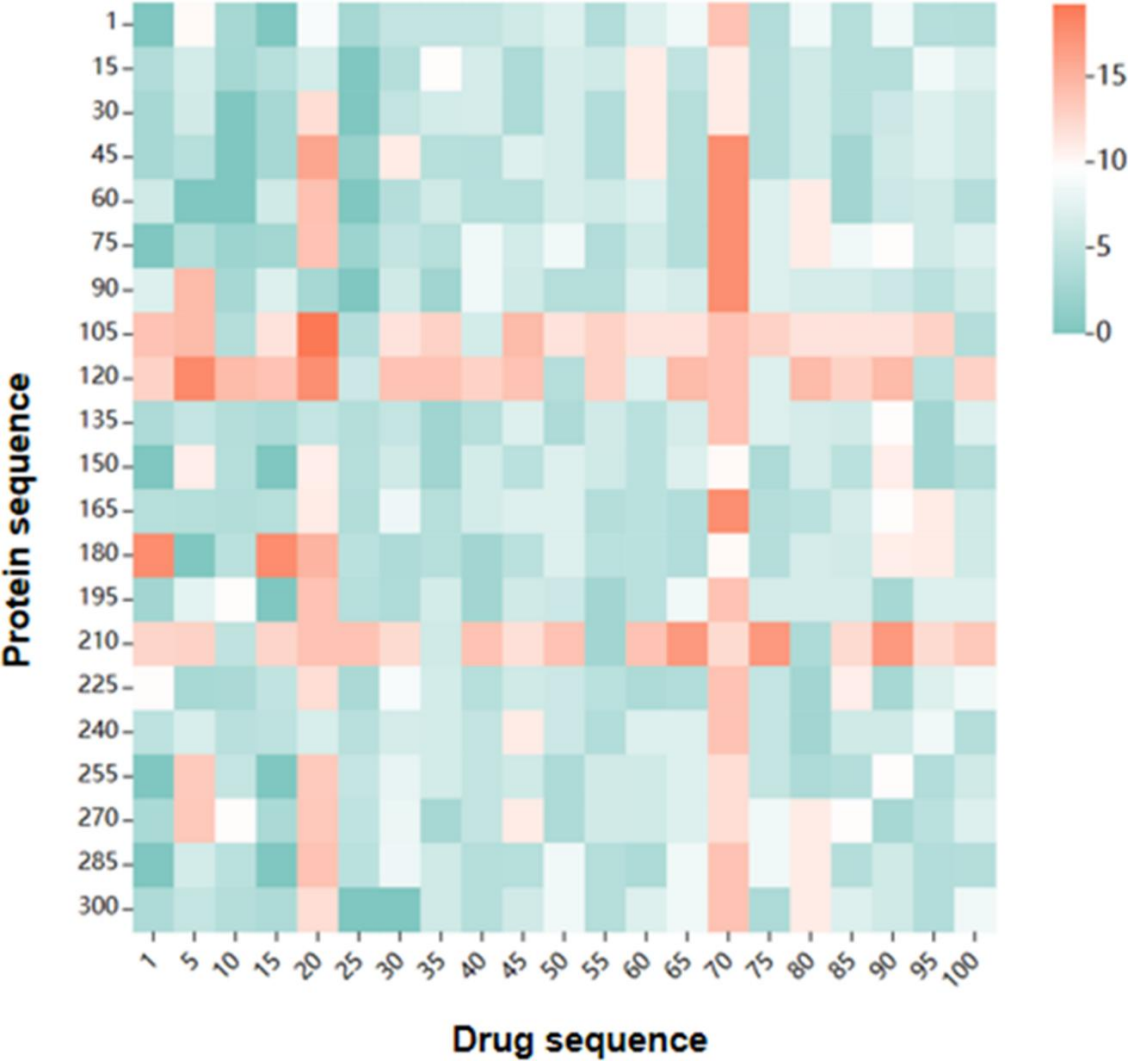
Visualization of sequence attention scores for 3C‐like proteinase (6WTK) and carbamic acid (GC373) predicted by the GCARDTI method.

The hybrid mechanism in GCARDT allows the model to measure the effect of the drug on the target protein as well as the effect of the protein on the drug in the interaction. To verify the interpretability of the model, 3C‐like proteinase (6WTK) and carbamic acid (GC373) were chosen, and attention mechanisms were found to account for the attention weights of protein feature vectors and the drug feature vector. Figure 5 shows the DTI scores of GC373 interacting with 6WTK. The figure shows target protein sequences clustered around the sequences at positions 105, 120, and 210 and the drug sequences around the atoms at positions 20 and 70.

**FIGURE 6:**
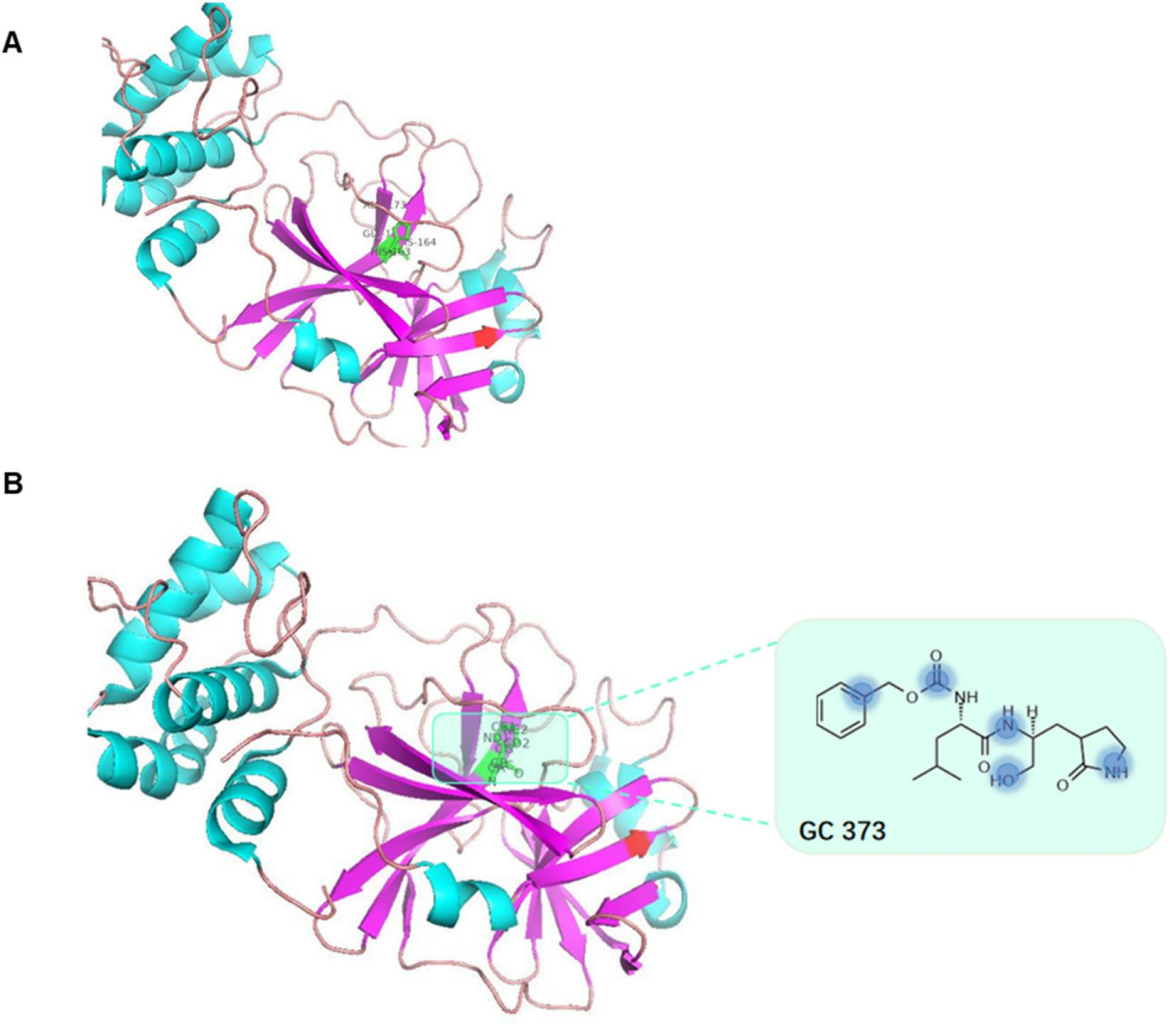
Visualization of the 6WTK binding site. Panel A highlights protein residues, and Panel B is a graphic representation of protein atoms and drug molecular structures.

To further validate this novel interaction and to verify the validity of the model, we performed computational docking and utilized the docking program AutoDock to infer the possible binding modes of the newly predicted DTIs. 3C‐like proteinase (6WTK) binds carbamic acid (GC373) by forming hydrogen bonds with residues ALA‐173, GLY‐174, HIS‐163, and HIS‐164. We use PyMOL for molecular docking and hydrogen bond coloring, and the atoms composed of these residues are combined with the atomic structure in the carbamic acid structure of the drug, as shown in Figure 6. PyMOL staining can intuitively indicate the binding site of the drug and the target. Binding sites can help researchers to not only pinpoint the 3D binding site of a protein but also allows identification of key residues and atoms, thus providing basic clues for drug development.

### 2.3 Number selection of random forest subtrees for predicting DTIs

**FIGURE 7:**
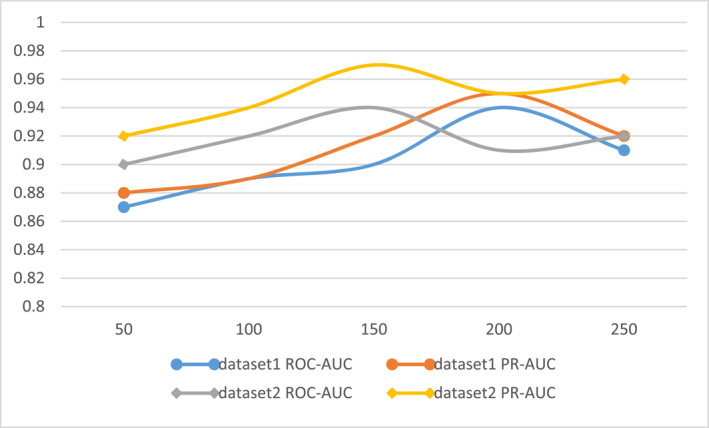
Effect of the number of subtrees of the random forest classification module of the GCARDTI method on the performance of the model.

In the classification module, the random forest module is used to process the features of drugs and target proteins. Random forest can judge the importance of features, determine the interactions between different features, and balance the error for imbalanced datasets. In this work, we ensured that the experiments were conducted under uniform and fair conditions. Different numbers of decision trees can significantly affect the performance of the model, and Figure 7 shows the effect of the number of neutron trees in the random forest on the performance of the model.

As can be seen from Figure 7, in dataset 1 with dot endpoints, the model performed best (ROC‐AUC = 0.94 and PR‐AUC = 0.95) when 200 random forest subtrees were used. In dataset 2 with square endpoints, the model performed best when 150 random forest subtrees were used. The model performance (ROC‐AUC = 0.94 and PR‐AUC = 0.97) was the best.

## 3 DISCUSSION

Precise identification of DTIs is one of the key steps in drug repositioning and drug development. In this study, we propose GCARDTI, a new model for predicting DTIs. The GCARDTI model uses the sequence structure of the target protein and the SELFIES of the drug to embed the atom and the sequence, respectively, so as to better extract the potential internal structure. Through the known association relationship, the heterogeneous network belonging to them can be constructed, so as to better extract the potential complex relationship between them. Its heterogeneous network is then fed into a hybrid mechanism consisting of the GCN and the GAT to capture high‐dimensional features from the medicinal chemical space and the target protein space. GCN generalizes the convolution operator to irregular regions. Specifically, it updates the atomic eigenvectors through their corresponding neighboring atoms connected by chemical bonds. This propagation mechanism automatically captures the structural information of molecules. The GAT attention module was used to capture the contribution of substructures to each drug and target. Finally, a layer of the GCN was used to aggregate the substructural information in each drug and target to update the feature vector of each drug and target and finally to obtain low‐dimensional and effective feature information on drug targets. In order to obtain better score prediction and avoid the negative impact of feature dimension and feature information importance on predicted drugs and targets, the random forest module was used to predict the score of feature representation, so as to improve the performance of DTI prediction. We used two datasets from different sources, and the results show that some indicators such as AUPR and AUROC of the GCARDTI model are better than other methods in some aspects. Our proposed GCARDTI is a drug SELFIE‐based GCN and GAT mixing mechanism that predicts drug target associations by integrating drug SELFIE‐based and protein sequences. GCARDTI provides an accurate and reliable source for drug discovery and drug repurposing and at the same time provides a new way of thinking in the field of DTI prediction and a drug SELFIES that differs from the traditional drug SMILES, opening up new methods of capturing drug target space.

### 3.1 Future and outlook

Although the GCARDTI model currently has good performance, it also has some potential drawbacks in extracting information from heterogeneous networks. Recently, as pointed out by Zhao et al. [41], existing computational models can only use low‐level biological information of individual drugs, diseases, targets, and their association levels. This germinates a new idea for future work, in which not only the multi‐source information on heterogeneous networks but also the high‐order meta‐path information should be integrated to improve the prediction performance and generalization performance of the model. At the same time, we also need to use more biological data sources, so that we can fully extract the potential feature of drugs and targets.

## 4 MATERIALS AND METHODS

### 4.1 Dataset

Two datasets were used in this paper, which were derived from the datasets used in previous studies; the datasets are linked to GitHub (see the end of this paper) and also downloaded from several public databases, DrugBank, UniProt, and MalaCard. DrugBank contains information on the molecular structure of drugs and target proteins. UniProt is a protein‐related database containing a large number of protein information. MalaCard is a human disease database that collects information about symptoms and related drug data. The two datasets involved a total of 1440 drugs and 3427 targets, 6901 known drug–target associations, 15,014 known drug–drug associations, and 12,341 known target–target associations, which are summarized in Table 5.

**TABLE 5:** Statistics of the GCARDTI dataset.

Dataset	Node types	Number	Edge types	Number
Dadaset 1	Drugs	708	Drug–target	1923
	Targets	1512	Drug–drug	10,036
			Target–target	7363
Dadaset 2	Drugs	732	Drug–target	4978
	Targets	1915	Drug–drug	15,014
			Target–target	12,341

### 4.2 Methods

#### 4.2.1 Drug and target protein similarity calculations

**FIGURE 8:**
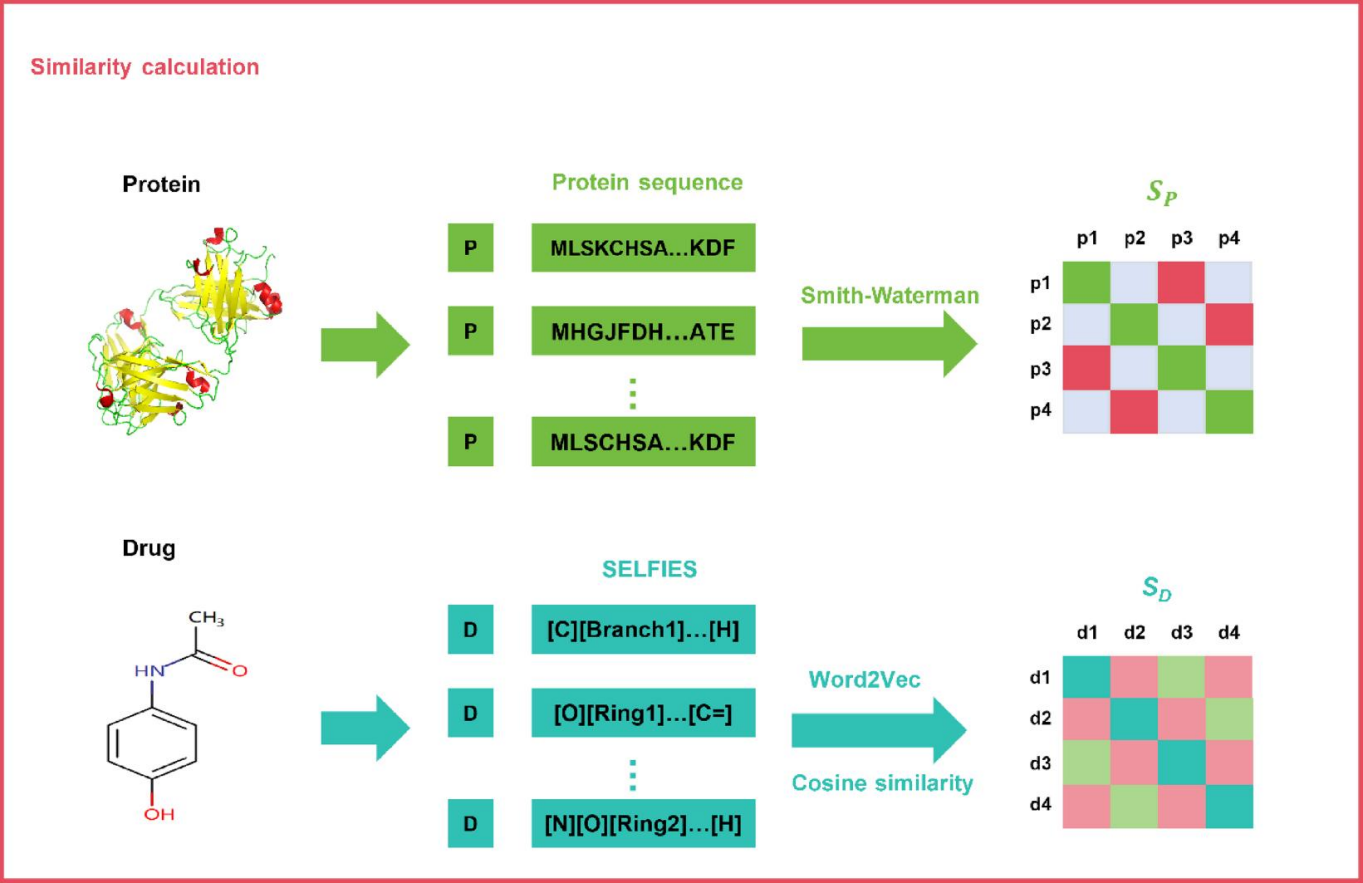
Calculation of drug and target protein similarity. The Smith–Waterman algorithm was used to calculate the similarity of the target protein sequence, and the target protein similarity matrix *S*_*P*_ was obtained. An autoencoder was used to extract the SELFIES of the drug, and cosine similarity was used to calculate the similarity of two drug SELFIES after Word2Vec atom embedding. The drug similarity matrix *S*_*D*_ was obtained.

The drug and target protein similarity calculation process is shown in Figure 8. Firstly, the target similarity calculation is introduced, using *P* = {*p*_1_, *p*_2_, *p*_3_, …, *p*_*m*_}, representing the *m* target proteins in the dataset. The similarity between the target protein sequence *p*_*i*_ and the target protein sequence *p*_*j*_ is calculated by using the Smith–Waterman algorithm [42] and then normalized by the following formula. Let the two sequences to be compared be *A* = *a*_1_ *a*_2_…*a*_*n*_ and *B* = *b*_1_ *b*_2_…*b*_*m*_ where *n* is the length of the sequences. In this paper, the maximum length of the protein sequence is 300, and the length beyond this length is truncated. *h*(*a*,*b*) is the similarity score between the elements that make up the sequence, *W*_*k*_ is the length, and *k* is the vacancy penalty, scoring from left to right and from top to bottom, filling the rest of the scoring matrix *SW*(*i*,*j*),

(1) $$SW\left(i,j\right)=\max\left\{\begin{array}{c} {SW}_{i-1,j-1}+h\left(a,b\right)\\ \max_{k\geq 1}\left\{{SW}_{i-k,j}-W_{k}\right\}\\ \max_{l\geq 1}\left\{{SW}_{i,j-l}-W_{l}\right\}\\ 0 \end{array}\right.\;\left(1\leq i\leq n,1\leq j\leq m\right),$$

where *SW*_*i*‐1,*j*−1_ + *h*(*a*,*b*) is the similarity score of *a*_*i*_, *b*_*j*_ is the alignment, max_*k*≥1_{*SW*_*i*‐*k*,*j*_−*W*_*k*_} is the score of *a*_*i*_ being at the end of a deleted section of length *k*, max_*l*≥1_{*SW*_*i*,*j*‐*l*_−*W*_*l*_} means the score of *b*_*j*_ at the end of the deletion of length l, 0 means *a*_*i*_, and *b*_*j*_ has no similarity up to this point. The procedure backtracks, starting from the element with the highest score in the matrix *SW*, and then backtracks to the previous position according to the source of the score; it keeps repeating until it encounters an element with a score of 0 and then normalizes it.

(2) $$S_{P}\left(i,j\right)=\frac{SW\left(i,j\right)-\min\;\left({SW}_{i}\right)\;}{\max\left({SW}_{i}\right)-\min\;\left({SW}_{i}\right)},$$

where *SW*(*i*,*j*) in the Formula (2) is said to use Smith–Waterman calculation target protein sequences between *p*_*i*_ and *p*_*j*_ score matrices; max(*SW*_*i*_) and $\min\;\left({SW}_{i}\right)$ represent the *p*_*i*_ and the other target protein between the highest and lowest score, respectively; the protein similarity matrix *S*_*P*_ ∈ *p*_*m*×*m*_ is then obtained by normalization of Equation (2).

The drug structure data is converted into the corresponding molecular structure SELFIES string by the string encoding algorithm of previous research, which can comprehensively and effectively show the drug molecular structure. Then, the atom embedding of SELFIES is carried out by Word2Vec, and the maximum length of the drug atom is 100, which is the same as the operation of protein sequence. The similarity between drugs is then calculated using cosine similarity, and the formula is as follows:

(3) $$S_{D}\left(i,j\right)=\frac{D_{i}^{r}\;\cdot {\;D}_{j}^{r}}{\left\|D_{i}^{r}\left\|\;\right\|D_{i}^{r}\right\|},S_{D}\left(i,j\right)\in \left[0,1\right],$$

where *v* drugs in the dataset were divided into *R* = {*r*_1_, *r*_2_, *r*_3_, …, *r*_*v*_}; $D_{i}^{r}$ represents the drug representation after embedding through Word2Vec atoms. The more similar the drugs are, the closer the value of *S*_*D*_(*i*,*j*) is to 1, and the drug similarity matrix *S*_*D*_ ∈ *R*_*v*×*v*_ is obtained.

#### 4.2.2 The heterogeneous network of drug and target protein was constructed

In order to better capture the potential association between various types of data and to better perform deep learning training on the common topological information on drug and target protein nodes, we constructed a heterogeneous network for drugs and target proteins, integrated the internal connections and mutual connections between different types, and captured the hidden biological information between each node. The similarity matrix of *S*_*D*_ and *S*_*P*_ and the correlation matrix of known target protein *A*_*DP*_ were used to construct *H*_*DP*_. The heterogeneous network contains two kinds of nodes *N* = {*N*_*D*_ ∪ *N*_*P*_} and three kinds of edges *E* = {*E*_*DP*_ ∪ *E*_*DD*_ ∪ *E*_*PP*_}. If there is a known association between two target proteins or drugs, there is a solid line edge between them in Figure 1. *E*_*DD*_ represents the edge between the drug and the drug; *E*_*DP*_ represents the edge between the drug and the target. Figure 1 shows a continuous line if there is an association between the drug and the target protein or a dotted line if it is not clear whether there is an association (Figure 1).

The adjacency matrix of the drug and target protein is represented as follows:

(4) $$H_{DP}=\left[\begin{array}{c} S_{D}\;{\;A}_{DP}\\ A_{DP}^{T}{\;S}_{P} \end{array}\right].$$

Integrating drug and target protein spatial information is based on the hybrid mechanism of the GCN and the GAT. The model uses a hybrid mechanism prediction that combines the GCN and the GAT to more effectively capture feature information from the drug and target space. For a drug or target protein node, the association and similarity associated with these can be regarded as the feature of the node, so the adjacency matrix of *H*_*DP*_ is regarded as the feature matrix *F*_*DP*_ of the drug or the target protein node.

#### 4.2.3 Node‐level feature capture—graph GCN

The GCN obtains feature information from the drug and target protein similarity network nodes and extends the convolution operator to irregular domains. Specifically, it updates atomic feature vectors by aggregating their corresponding chemically bonded neighborhood atoms. Given a network G, the input feature matrix is *F*_*DP*_ ∈ *R* ^*N*×*F*^, the adjacency matrix is *A*_*DP*_ ∈ *R* ^*N*×*N*^, *N* is the number of drug or target protein nodes, and *F* is the characteristic dimension of nodes. Here, we set the maximum number of atoms of the drug to 100 and the maximum length of the protein sequence to 300; we fill 0 if the data is not enough and crop if it is out of range. The formula is as follows:

(5) $$F_{DP}^{\left(l+1\right)}=GCN\left(H_{DP},{\;F}_{DP}^{\left(l\right)}\right)=\sigma \left(D^{-\frac{1}{2}}\tilde{{\;H}_{DP}\;}D^{-\frac{1}{2}}{\;F}_{DP}^{\left(l\right)}W^{\left(l\right)}\right),$$

where *D* is the degree matrix of the input graph, $\tilde{{\;H}_{DP}\;}=I+{\;H}_{DP}$ is the adjacency matrix with self‐connections, ${\;F}_{DP}^{\left(l\right)}$ is the *l*th‐layer embedding of the input nodes, *W* ^(*l*)^ is the *l* layer trainable weight matrix, and *σ* is the nonlinear activation function RELU.

#### 4.2.4 Local module feature capture—graph attention network

**FIGURE 9:**
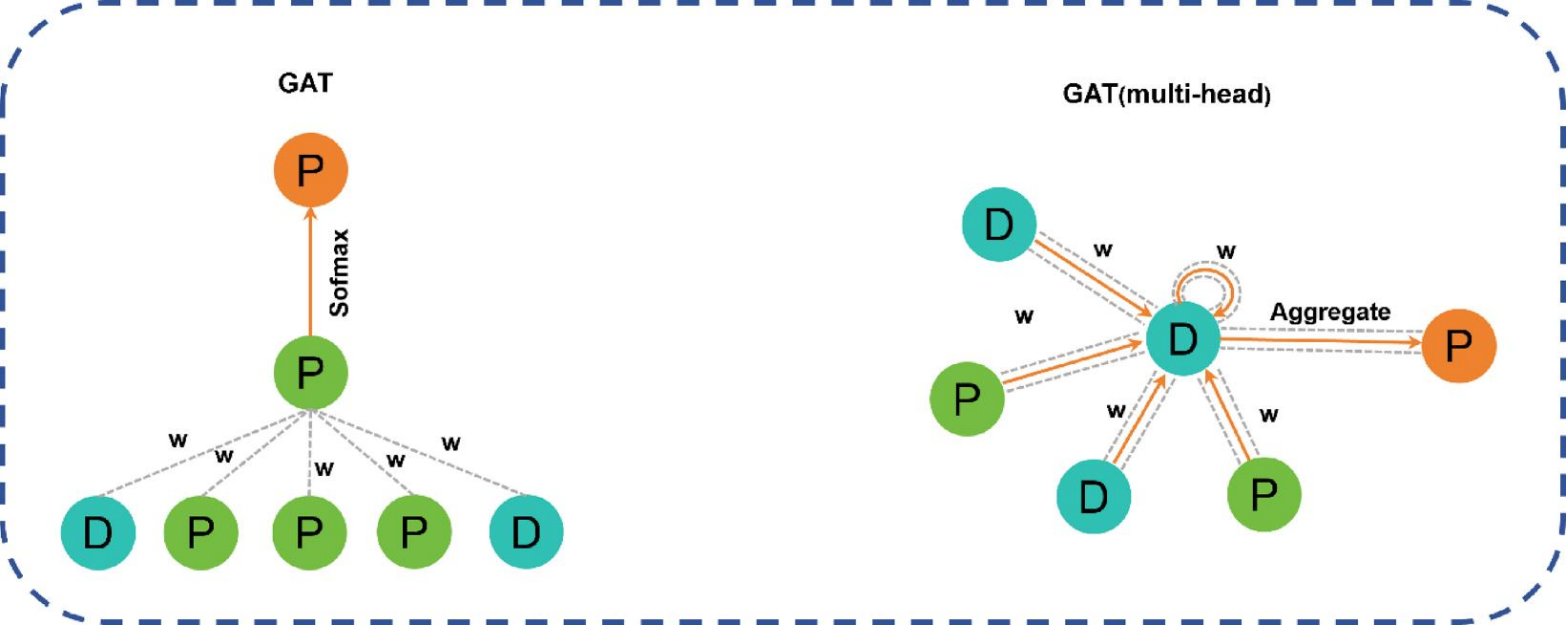
GAT workflow. (1) Calculation of the attention coefficient. For the left side of the figure, firstly, linear transformation is performed on the two nodes, and the red node P then calculates its neighbor nodes and its correlation coefficient one by one. (2) Weighted summation for each node GAT output incorporates new features of its domain information. The right side of the figure shows the use of multi‐head attention, which establishes different mapping information in multiple different mapping spaces, use multiple attention modules, operates on the graph, and finally integrates the results.

The GAT assigns different weights to neighboring nodes according to their importance in the process of information aggregation of central nodes. Here, the GAT receives the output from the previous GCN step to capture the contribution of local substructures to each drug and target protein. In this paper, multi‐head attention mechanism is used to capture its features (Figure 9.), so that our model pays more attention to important nodes and reduces the influence of edge noise.

For the network G, the GAT outputs the following,

(6) $$F_{DP}^{\left(l+n\right)}=GAT\left(H_{DP},{\;F}_{DP}^{\left(l+1\right)}\right),$$

(7) $$F_{i}^{new}=\sigma \left(\frac{1}{K}\sum_{k=1}^{K}\sum_{j\neq i}\emptyset_{ij}^{k}W_{k}F_{i}^{\left(l+n\right)*}\right),$$

(8) $$\emptyset_{ij}^{k}=\frac{\exp\;\left(LR\left(a_{k}^{T}\left[W_{k}F_{i}^{\left(l+n\right)*}∥W_{k}F_{j}^{\left(l+n\right)*}∥B_{k}S_{ij}\right]\right)\right)}{\sum_{t\neq i}\exp\left(LR\left(a_{k}^{T}\left[W_{k}F_{i}^{\left(l+n\right)*}∥W_{k}F_{t}^{\left(l+n\right)*}∥B_{k}S_{it}\right]\right)\right)},$$

In the formula, $F_{i}^{new}$ is the feature representation of node *i* in $F_{DP}^{\left(l+n\right)}$; ${\;F}_{i}^{\left(l+n\right)*}$ is the input feature of node *i* of drug or target protein; $\emptyset_{ij}^{k}$ is the *K* attention coefficient between node *i* and node *j*; and vector *K* is the number of attention heads. *W*_*k*_ is the weight matrix of the *K* attention mechanism; ∥ is the connection operator; *LR* is the LeaklyReLU activation function; $a_{k}^{T}$ is the weight vector of the *K* attention mechanism. *B*_*k*_ is the weight of the edge *S*_*ij*_ to be learned.

Through GAT’s feature capture, the GCN is used again to aggregate the new round of structural information on nodes in each drug and target protein to update the feature vectors of each drug and target protein, and finally the feature representations *X*_*D*_ and *X*_*P*_ of the drug and target protein are obtained. The drug and target protein representations are spliced to obtain the drug–target fusion representation pair *Z*_*X*_,

(9) $$Z_{X}=\left[X_{D},X_{P}\right].$$

In this paper, in order to obtain better score prediction and avoid the negative effects of feature dimension and importance of feature information on predicting drugs and targets, a random forest classifier was used. Random forest is an ensemble algorithm composed of decision trees, which uses multiple decision trees to alleviate the problem of overfitting the training data. It can judge the importance of features, can judge the mutual influence between different features, and can balance the error for imbalanced datasets. Random forest has strong robustness and generalization ability and has been widely used to solve prediction problems in the field of bioinformatics.

1. The first step is to sample the data in the training set in the form of putting back, sampling the dataset *N* times, and training *N* CART (Classification and Regression Tree) decision trees.
2. Then, the Gini coefficient is used to calculate the optimal segmentation variable, and the decision tree is constructed by splitting the node attributes.
(10) $${Gini}_{index\left(x,f\right)}=\sum_{V=1}^{V}\frac{\left|X^{v}\right|}{\left|X\right|}Gini\left(X^{v}\right),$$
(11) $$Gini\left(X^{v}\right)=1-\sum_{i=1}^{\left|y\right|}p_{i}^{2}\;.$$
  where *X* is the sample set; *p*_*i*_ is the proportion of the *i* th classification in *X*; *X* ^*v*^ is the sample set of *X* with the *v* value of *f*; and *f* is the set of feature attributes. We used the low‐dimensional feature representation *Z*_*X*_ obtained by autoencoder training as input. In the training stage, pairs of drugs and targets formed the training set and were then input to the random forest. Finally, the DTI prediction score matrix was obtained.
3. By repeating the previous steps *N* times to obtain *N* decision trees, the drug–target association was predicted according to the results of the decision trees.

Finally, all learnable parameters were jointly optimized by back propagation. The training objective was to minimize the cross‐entropy loss, as follows:

(12) $$L=-\sum_{i}\left(y_{i}\;\log\left(p_{i}\right)+\left(1-y_{i}\right)\log\left(1-p_{i}\right)\right)+\frac{\lambda }{2}∥\theta {∥}_{2\;}^{2},$$

where *θ* is the set of all the above learnable weight matrices and bias vectors; *y*_*i*_ is true labels; *p*_*i*_ outputs probabilities; and *λ* is the hyperparameter of *L*2 regularization (Algorithm).

The process of GCARDTI is fully described in the algorithm as follows:

**The complete procedure of GCARDTI.**

Input: graph *H*_*DP*_(V, A, and E)
 the number of drugs: *n*
 the number of proteins: *m*
 the number of trees: *t*
Output: the relationships matrix ${P\in R}^{E^{DP}}$ of node *V*_*i*_ and node *V*_*j*_ *V*_*i*_, *V*_*j*_ ∈ *V*
1: Initialization: **P**
2: Calculate the chemical structure similarity information on drugs *S*_*D*_ ∈ *R*_*n*×n_
3: Calculate the sequence similarity information on proteins $P_{\in }P_{m\times m}$
4: Building heterogeneous networks *H*_*DP*_
5: Capture node feature information
6: $F_{DP}^{\left(l+1\right)}=GCN\left({\;H}_{DP},{\;F}_{DP}^{\left(l\right)}\right)$
7: $F_{DP}^{\left(l+n\right)}=GAT\left({\;H}_{DP},{\;F}_{DP}^{\left(l+1\right)}\right)$
8: $X_{D},X_{P}=GCN\left({\;H}_{DP},{\;F}_{DP}^{\left(l+n\right)}\right)$
9: *Z*_*X*_ = [*X*_*D*_,*X*_*P*_]
10: P = Random Forest Classifier (*Z*_*X*_, t)
11: end for
12: Predicted unknown drug–target associations in **P**

## AUTHOR CONTRIBUTIONS

Yinfei Feng: performed the experiments, analyzed the data, and wrote the paper. Yuanyuan Zhang: provided ideas for the article and reviewed the manuscript. Zengqian Deng and Mimi Xiong provided the source of the data. All authors have approved the final version of the article.

## CONFLICT OF INTEREST STATEMENT

The authors Yinfei Feng, Yuanyuan Zhang, Zengqian Deng, and Mimi Xiong declare that they have no conflict of interest or financial conflicts to disclose.

## ETHICS STATEMENT

All procedures performed in studies involving animals were in accordance with the ethical standards of the institution or practice at which the studies were conducted and with the 1964 Helsinki declaration and its later amendments or comparable ethical standards.

## Supporting information

Supplementry material S1

## Data Availability

All instructions and code for our experiments can be found on the GitHub website (FengYinFei/GCARDTI).
