# Supplementary material for: GCARDTI: Drug–target interaction prediction based on a hybrid mechanism in drug SELFIES
Source: Quant Biol. 2024 Apr 1;12(2):141–54. doi: 10.1002/qub2.39 (PMC12806471; doi:10.1002/qub2.39)
Supplement: Supplementary file 1 — Supplementry material S1 [file QUB2-12-141-s001.pdf]

Supplementary material

To better evaluate the performance of the model, we decided to use the accuracy of the first k DTIs (10%, 20%, 50%, 100%). The accuracy rate can reflect whether the model can reasonably predict the performance of DTIs. We still select the accuracy of these methods to compare the performance of our methods, as shown in Figure 1.

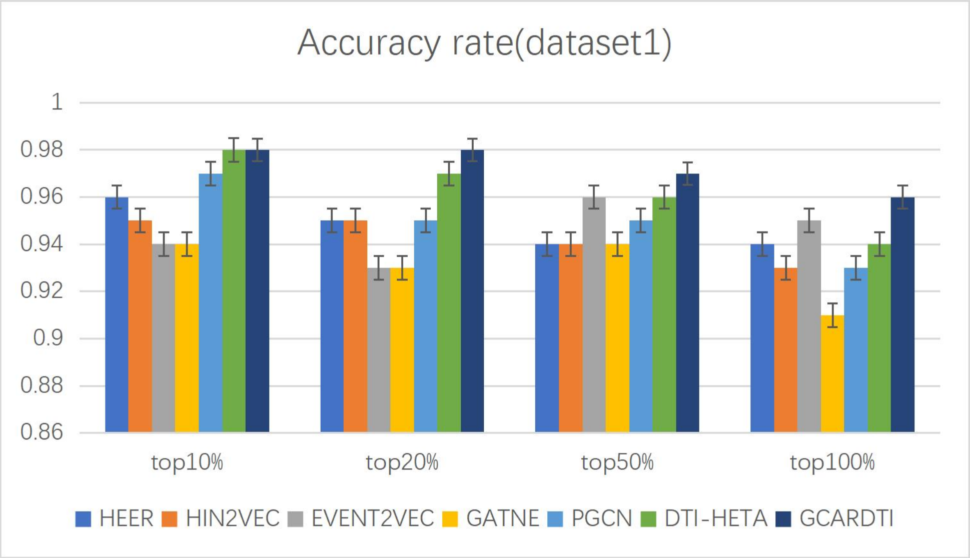

Figure 1. Comparison of the accuracy of GCARDTI method and the other six methods in dataset 1.

As can be seen from FIG. 1, DTI-HETA and our model are the best. In the top10%, their model performance is the same, both are 0.98, while in the latter (20%, 50%, 100%) GCARDTI model performance is better than the previous several algorithms. Similarly, using the second data set in Figure 2, our model outperforms several other algorithms. The GCARDTI model can better capture characteristic information from the drug and target space.

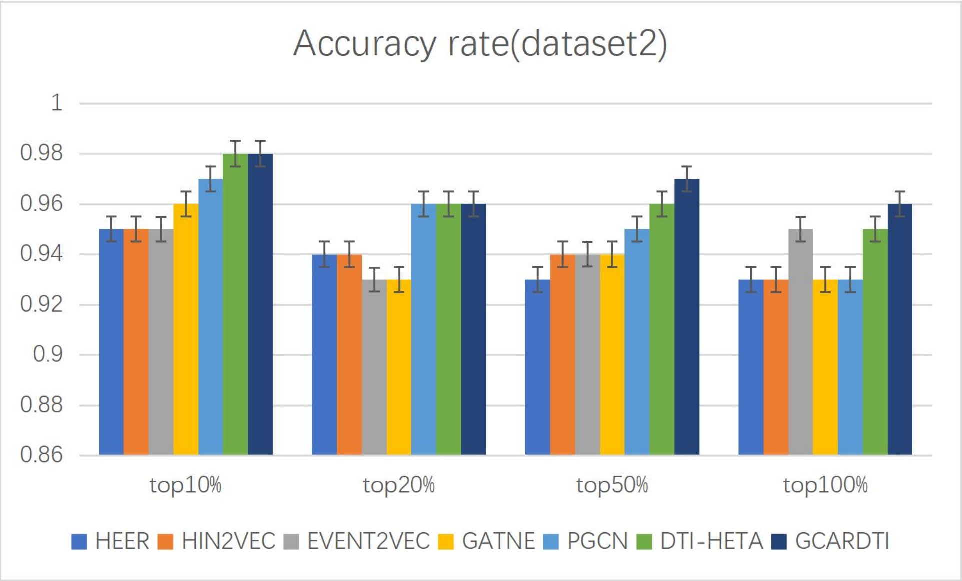

Figure 2. Comparison of the accuracy of GCARDTI method and the other six methods in dataset 2.
